# Seasonal Variations and Trends in Hospitalization for Peptic Ulcer Disease in the United States: A 12-Year Analysis of the Nationwide Inpatient Sample

**DOI:** 10.7759/cureus.854

**Published:** 2016-10-30

**Authors:** Ritesh Kanotra, Moiz Ahmed, Nileshkumar Patel, Badal Thakkar, Shantanu Solanki, Sarah Tareen, Matthew J Fasullo, Mayurathan Kesavan, Nikhil Nalluri, Ahsan Khan, Dhaval Pau, Liliane Deeb, Jeffrey Abergel, Ananya Das

**Affiliations:** 1 Department of Internal Medicine, Banner Baywood Medical Center, Mesa, Arizona, USA; 2 Department of Internal Medicine, Staten Island University Hospital; 3 Department of Cardiology, University of Miami Miller School of Medicine/Jackson Memorial Hospital, Florida, USA; 4 Department of Internal Medicine, Rutgers New Jersey Medical School; 5 Department of Internal Medicine, Westchester Medical Center at New York Medical College, Valhalla, NY, USA; 6 Institute of Clinical Research, India; 7 Medicine, UMass Memorial Medical Center; 8 Department of Gastroenterology, Staten Island University Hospital; 9 Professor of Medicine, Creighton University; 10 Chief of Gastroenterology, St. Joseph Hospital Medical Center

**Keywords:** peptic ulcer disease, seasonal variation, national trends, hospitalization cost, nis, icd-9, los, coh, gastric ulcer, duodenal ulcer

## Abstract

**Background:**

Peptic ulcer disease (PUD) is a major public health burden significantly impacting the cost of hospitalization in the United States (US). We examined the trends, characteristics, complications, cost, and seasonality of PUD-related hospitalizations from 2000 to 2011.

**Methods:**

With the use of the Nationwide Inpatient Sample from 2000 through 2011, we identified PUD-related hospitalizations using the International Classification of Diseases (ICD-9), 9th Revision, and the Clinical Modification code 531.00 to 534.91 as the principal discharge diagnosis. The total number of hospitalizations for each calendar month of the year were added over a 12-year period, and this number was divided by the number of days in that particular month to obtain the mean hospitalizations per day for each month.

**Results:**

The study found that 351,921 hospitalizations with the primary discharge diagnosis of peptic ulcer disease (PUD) occurred in the US between 2000 and 2011. This number dropped significantly from 49,524 to 17,499 between 2000 and 2011, and the rate of PUD-related mortality decreased from 4.3% to 3.1%. The mean age of the study population was 66.2 ± 17.4 years; 52.3% were males, and 56.8% were white. The number of hospitalizations in the US peaked in the spring season (916/day), and reached a nadir in the fall season (861/day). The mean cost of PUD hospitalization increased significantly from $11,755 in 2001 to $13,803 in 2011 (relative increase of 17%; p <0.001).

**Conclusion:**

The incidence of PUD and its mortality has decreased significantly in the last decade, but its economic burden on the healthcare system remains high. A seasonal pattern of PUD hospitalization showed a peak in PUD-related admissions in the spring season and a trough in the fall season.

## Introduction

Peptic ulcer disease (PUD) and its complications affect about six million individuals per year in the US, contributing fairly to increased healthcare costs [1]. PUD exerts a significant economic impact directly, in terms of hospital costs and indirectly by the significant loss of workdays. Since the advent of effective antimicrobial agents in the 1980s [2] and increased awareness about PUD association with Helicobacter pylori (H. pylori) since 1990s [3], there has been a downward trend in the H. pylori-related PUD hospitalizations in the US. Interestingly, there has been an increase in the incidence of non-H. pylori and non-NSAID-related peptic ulcers in the United States, accounting for about 30% of ulcers observed in the region [4]. This indicates that the incidence of PUD may also be related to other factors such as age, sex, geographical distribution, seasonal trend, lifestyle, and genetics besides H. pylori, its primary cause.

Some other studies conducted have shown increased incidence of peptic ulceration in the elderly, men, and in people with dietary habits involving spicy food or increased salt intake and also increased smoking and alcohol consumption [4].

The seasonal periodicity in the occurrence of ulcer disease has been described numerous times previously in other countries, with most studies describing winter abundance and a summer deficit [5-7]. Some studies showed two peaks in spring and autumn [8]; whereas a large scale study in Italy by Roberto Manfredini, et al. [9] described three peaks in spring, autumn and winter. Various other disease processes like myocardial infarction, cerebrovascular accident and congestive heart failure have shown seasonal variation in their hospitalization rates [10-11]. There has been little evidence to confirm the impact of seasons and also the regional distribution on PUD in the US, and also there is limited information with regard to the cost of healthcare and the length of stay for PUD in the US.

We aim to ascertain a seasonal periodicity of PUD hospitalization by looking into the largest database available in the US and also determine the cost of hospitalization and length of stay.

## Materials and methods

### Data source

The National Inpatient Sample (NIS) was used to obtain data from 2000 to 2011. This is the largest available inpatient all-payer inclusive registry available in the US, which includes approximately seven to eight million discharges per year [12]. This sample is designed to represent approximately 20% of US community hospitals. National estimates were calculated using sampling weights provided by the NIS. Each individual entry consists of demographic information, which includes age, sex, ethnicity, insurance and socioeconomic status, comorbidities, hospitalization outcome, length of stay, and the cost of hospitalization. The NIS database contains one primary discharge diagnosis and up to 24 secondary discharge diagnoses during the period of hospitalization. The severity of comorbid conditions was defined using Deyo modification of Charlson comorbidity index, calculated using ICD-9 codes. The NIS data has been used previously to study national trends of several diseases, medical procedures and their complications, and health care usage [13].

This study was exempt from IRB review after human subject research determination. 

### Study population

Utilizing the NIS data from 2000 through 2011, PUD-related admissions were identified using the International Classification of Diseases (ICD-9), 9th Revision, Clinical Modification code 531.00 to 534.91 as the principal discharge diagnosis. Using the same codes, the location of the ulcer (gastric, duodenal, or others) and PUD-related complications like hemorrhage, perforation and hemorrhage with perforation were also identified. Patients <18 years of age, admissions with missing age, sex, race, and admission or discharge dates were excluded.

### Outcomes

The primary goal of our study was to determine the trends in the rate of hospitalization for PUD, its complications based on the ulcer location, and the demographic characteristics of the PUD population. The secondary goal of our study was to determine the economic burden of PUD in the health care system by evaluating the average cost per hospitalization and the length of stay and to delineate the seasonality of PUD in the US and its various regions over the 12-year period.

### Definition of variables

The NIS variables were used to identify patient age, sex, and race. Race was divided into White, Black, Hispanic and others. Age was divided into five groups: 18–34 years of age, 35–49 years of age, 50–64 years of age, 65–79 years of age, and 80 years of age or older. PUD-related complications were divided into hemorrhage, perforation, or hemorrhage with perforation. Their sites of occurrence were gastric, duodenal or unknown. We considered participating hospitals as teaching hospitals only if they had an Accreditation Council for Graduate Medical Education (ACGME) accredited residency program, were a member of the Council of Teaching Hospitals, and/or had full-time equivalent interns and a resident to patient ratio of >=0.25. Hospital location (rural/urban) and bed size were also taken into account. The length of stay (LOS) for each hospitalization was calculated after excluding those who died during their stay. Cost of hospitalization (COH) was determined after merging data with cost-to-charge ratio files available from the Healthcare Cost and Utilization Project (HCUP) website. The total cost of each hospitalization was determined by multiplying the cost-to-charge ratio with the total hospital charge. Inflation was accounted for by adjusting the cost of each year in reference to the 2011 US dollar value using Consumer Price Index data. The regions of the US were divided as Northeast (NE), Midwest (MW), South (S), and West (W). The four seasons were divided as spring (March to May), summer (June to August), fall (September to November) and winter (December to February).

### Statistical analysis

SAS 9.4 (SAS Institute Inc, Cary, North Carolina) was used for the analyses. Nationally representative estimates were produced using the weight variable provided by the NIS. The categorical variables are expressed in terms of the percentage (%) of the total study population whereas continuous variables are expressed in terms of mean with its standard error. The cost for each year was calculated in terms of the 2011 cost, after adjusting for inflation according to the latest consumer price index (CPI) data released by US Government. For trend analysis, the Cochran-Armitage test for categorical variables and linear regression for continuous variables was utilized. A P-value of <0.05 was considered statistically significant. To identify the significant predictors of length of stay and cost, we generated two-level hierarchical mixed effects linear regression models (with patient-level factors nested within hospital-level factors) with the unique hospital identification number incorporated as random effects within the model. In each of these multivariate models, patient-level and hospital-level variables: age (per 15 years change), gender, Deyo modification of Charlson comorbidity index, primary payer, median household income category (as per patient’s residential zip code), admission type (elective vs. nonelective), weekend admissions, hospital beds-size category (as per hospital’s location and number of beds), hospital region (Midwest, South or West with Northeast as referent), and the hospital’s teaching status (teaching vs. nonteaching) were included. The frequency of hospitalization was calculated per month over a cumulative 12-year (2000–2011) period, and then this number was divided by the number of days in the month to calculate the mean hospitalizations per day for each month.

## Results

### Demographics

We identified a total of 351,921 hospital admissions with the primary discharge diagnosis of PUD, of which 54% were gastric in location, 43% duodenal and the remaining three percent unspecified site. Table 1 shows baseline characteristics of the study population from 2000 to 2011. The number of PUD hospitalizations decreased from 49,524 in 2000 to 17,499 in 2011. The mean age of the study population was 66.2 ± 17.4 years (mean ± SD); 52% were males and 48% were females. The majority of the patients were White (57%). Hospital admissions for PUD were more common in patients 65–79 years of age (33%); most had Medicare (58%) as the primary payer, the majority of those admissions occurred emergently (89%), and most involved hospitals were located in an urban nonteaching setting (47%). There was no significant difference in the overall admissions for PUD based on the median household income. The mean cost during the study period was $13,324 (Std. error=87), and the mean length of stay was six days (Std. error=0.02).

**Table 1:** Baseline characteristics

Year	2000	2001	2002	2003	2004	2005	2006	2007	2008	2009	2010	2011	Overall	P-value
Frequency	49524	45983	36597	35769	33177	28902	26636	22071	20261	18541	16961	17499	351921	
Age (years %)														
18-34	4.6	4.3	4.5	4.2	4.5	4.8	4.6	5.3	4.4	5.3	5.1	5.3	4.6	<.0001
35-49	13.7	13.2	13.5	12.6	13.1	13.1	12.7	13.1	12.9	11.5	12.3	11.3	12.9	<.0001
50-64	20.0	19.9	21.0	22.7	22.2	23.4	24.7	26.1	26.1	24.6	28.1	25.2	22.8	<.0001
65-79	34.4	34.8	33.3	33.0	32.5	32.4	32.3	29.5	30.2	32.4	30.1	31.1	32.7	<.0001
>=80	26.8	27.4	27.2	27.0	27.0	25.4	25.1	25.2	25.8	25.8	23.6	26.5	26.4	<.0001
Gender (%)														<.0001
Male	53.2	52.04	52.2	50.2	50.1	52.8	52.1	53.8	52.7	54.5	54.2	51.5	52.3	
Female	46.8	48.0	47.8	49.7	49.7	47.2	47.8	46.0	47.2	45.4	45.8	48.5	47.7	
Race (%)														
White	58.8	55.4	53.2	54.0	54.4	56.3	52.9	52.9	58.6	64.6	63.2	68.9	56.8	<.0001
Black	7.3	7.4	7.9	7.7	8.7	7.0	7.9	8.9	9.1	8.2	12.4	11.0	8.3	<.0001
Hispanic	4.6	4.6	5.1	6.9	5.7	6.1	6.7	5.2	5.4	6.7	7.4	7.3	5.7	<.0001
Others	5.17	4.7	5.72	5.47	5.32	6.31	6	6.5	6.3	7.1	7.4	5.6	5.8	<.0001
Missing	24.1	27.9	28.1	25.9	24.4	26.5	26.6	20.5	13.4	9.6	7.1	4.7	22.61	
Primary Payer (%)														
Medicare	59.1	59.6	59.5	60.3	58.8	59.1	57.0	53.7	55.9	57.6	53.4	58.3	58.2	<.0001
Medicaid	5.4	6.4	6.4	7.6	8.0	6.9	6.6	8.3	8.0	6.9	10.0	7.9	7.1	<.0001
Private	26.9	26.3	25.3	23.7	24.2	24.3	25.9	26.7	25.7	25.7	25.6	23.2	25.4	<.0001
No-pay/Self-pay	8.1	7.6	8.6	8.3	8.8	9.6	10.3	11.1	10.2	9.6	10.9	10.2	9.1	<.0001
Hospital Region (%)														
Northeast	18.1	18.7	20.7	19.2	20.5	25.3	20.7	21.3	16.0	20.2	19.5	21.5	20.0	<.0001
Midwest or North Central	24.4	21.4	20.9	20.9	24.6	20.2	17.8	16.8	19.1	15.8	16.2	19.6	20.6	<.0001
South	33.5	32.2	28.7	30.3	33.9	33.0	29.1	27.6	30.5	28.7	26.0	26.8	30.7	0.0071
West	24.0	20.2	21.0	20.7	21.0	21.6	19.9	20.1	19.6	19.2	21.1	16.0	20.8	0.0017
Missing	0.0	7.5	8.8	8.9	0.0	0.0	12.5	14.3	14.8	16.0	17.1	16.1	8.0	
Hospital Teaching status (%)														
Rural	22.6	23.6	20.6	25.3	21.6	21.2	18.9	17.6	17.9	17.2	16.3	16.7	20.8	<.0001
Urban non-teaching	47.1	46.4	47.3	45.1	48.9	46.6	43.7	50.1	48.6	46.8	45.7	45.5	46.8	0.5391
Urban teaching	30.2	30.0	32.1	29.6	29.5	32.2	36.5	32.3	33.5	35.4	37.2	36.8	32.2	<.0001
Admission types (%)														<0.0001
Emergent/Urgent	76.7	79.4	92.2	91.9	91.2	91.8	93.2	93.8	94.5	95.0	94.3	94.1	88.84	
Elective admission	8.8	7.94	7.58	7.8	7.89	8.06	6.59	6.07	5.4	5.0	5.7	5.71	7.28	
Median Household Income (%)														
1. 0-25th percentile	6.12	6.09	4.71	26.5	29.3	27.3	27.4	27.7	28.5	26.1	27.6	27.6	19.4	<.0001
2. 26-50th percentile	31.7	24.6	21.1	30.0	27.3	26.5	26.6	25.8	28.4	26.9	27.0	26.2	27.0	0.0003
3. 51-75th percentile	26.8	28.4	25.7	22.8	21.7	23.0	19.9	21.4	21.9	21.7	23.3	24.5	24.0	<.0001
4. 76-100th percentile	33.5	39.7	45.7	18.3	19.7	21.0	22.8	22.3	19.2	22.9	18.9	19.8	27.4	<.0001
In-hospital mortality (%)	4.3	4.7	4.2	4.3	4.3	4.01	4.0	3.7	3.2	3.6	3.3	3.1	4.0	<.0001
Cost ($)														
Mean (std. error)	-	11,755 (216)	12,706 (261)	13,287 (277)	13,052 (244)	13,539 (290)	13,674 (298)	13,919 (312)	13,652 (319)	14,415 (341)	15,269 (391)	13,803 (338)	13,324 (87)	<0.001
LOS (days)														
Mean (std. error)	5.8 (0.07)	5.9 (0.07)	6.0 (0.09)	6.0 (0.09)	5.7 (0.08)	5.8 (0.09)	5.8 (0.10)	6.0 (0.12)	6.0 (0.13)	6.0 (0.12)	6.0 (0.13)	5.6 (0.10)	5.9 (0.02)	0.983

The mean cost of PUD hospitalization increased significantly from $11,755 in 2001 to $13,803 in 2011 (relative increase of 17%; p <0.001). Mean cost of PUD hospitalization for the year 2000 was not available. Our multivariate hierarchical linear regression model, as depicted in Table 2, shows a lower cost of care by $1,616 for patients with private insurance as compared to patients with Medicare or Medicaid being the primary payer (p <0.0001). Large bedded teaching hospitals cost $2,011 more than small nonteaching hospitals, while the urban teaching hospitals cost $2,456 more than the rural or urban nonteaching hospitals. The regional cost variation with Northeast as the reference shows Midwest and Southern regions to be less expensive by $2,571 and $2,557, respectively (p< 0.0001).

**Table 2:** Multivariate hierarchical linear regression model to identify significant predictors of cost of care for PUD-related hospitalizations

Variables	Beta-coefficient ($)	Std. Error of Beta ($)	LL of 95% CI of Beta ($)	UL of 95% CI of Beta ($)	P-value
Age (per 10 years increase)	89	68	-43	222	0.187
Sex					
Female	-174	191	-549	200	0.362
Male	Referent				
Charlson score					.
0-1	Referent				
2	3,287	216	2,863	3,711	<.0001
>=3	6,044	288	5,479	6,609	<.0001
Primary Payer					.
Medicare or Medicaid	Referent				
Private including HMOs & PPOs	-1,616	257	-2,120	-1,112	<.0001
Other/Self-pay/No charge	-1,073	370	-1,799	-347	0.004
Median Household Income Category for patient's Zip code					.
1. 0-25th percentile	Referent				
2. 26-50th percentile	-362	285	-921	198	0.205
3. 51-75th percentile	-20	300	-607	567	0.947
4. 76-100th percentile	192	315	-426	809	0.543
Admission Type					
Non-elective	Referent				
Elective	2,133	383	1,383	2,884	<.0001
Admission Day					
Weekdays	Referent				
Weekends	-107	27	-160	-53	<.0001
Bed size of Hospital depending on Location & Teaching Status					
Small	Referent				
Medium	1,610	387	853	2,368	<.0001
Large	2,011	366	1,294	2,728	<.0001
Hospital Region					
Northeast	Referent				
Midwest	-2,571	431	-3,415	-1,727	<.0001
South	-2,557	419	-3,378	-1,735	<.0001
West	206	458	-692	1,104	0.653
Hospital Location & Teaching Status					
Rural or Urban Non-teaching	Referent				
Urban Teaching	2,456	301	1,866	3,045	<.0001

There was no significant difference in the mean length of stay from 5.8 days in 2000 to 5.6 days in 2011 (p = 0.983). The predictors of length of stay are shown in Table 3.

**Table 3:** Multivariate hierarchical linear regression model to identify significant predictors of length of stay for PUD-related hospitalizations

Variables	Beta-coefficient ($)	Std. Error of Beta ($)	LL of 95% CI of Beta ($)	UL of 95% CI of Beta ($)	P-value
Age (per 10 years increase)	0.15	0.02	0.11	0.19	<.0001
Sex					
Female	0.16	0.06	0.04	0.27	0.009
Male	Referent	.			.
Charlson score					
0-1	Referent				
2	1.12	0.07	0.98	1.25	<.0001
>=3	2.10	0.09	1.93	2.28	<.0001
Primary Payer		.			.
Medicare or Medicaid	Referent				
Private including HMOs & PPOs	-0.89	0.08	-1.05	-0.73	<.0001
Other/Self-pay/No charge	-0.56	0.12	-0.79	-0.33	<.0001
Median Household Income Category for patient's Zip code		.			
1. 0-25th percentile	Referent				
2. 26-50th percentile	-0.21	0.09	-0.38	-0.03	0.021
3. 51-75th percentile	-0.10	0.09	-0.28	0.09	0.297
4. 76-100th percentile	-0.09	0.10	-0.28	0.10	0.334
Admission Type					
Non-elective	Referent				
Elective	0.48	0.12	0.25	0.70	<.0001
Admission Day					
Weekdays	Referent				
Weekends	-0.04	0.01	-0.06	-0.02	<.0001
Bed size of Hospital depending on Location & Teaching Status					
Small	Referent				
Medium	0.74	0.11	0.53	0.96	<.0001
Large	0.97	0.10	0.77	1.17	<.0001
Hospital Region					
Northeast	Referent				
Midwest	-1.02	0.12	-1.25	-0.79	<.0001
South	-0.52	0.11	-0.73	-0.30	<.0001
West	-1.00	0.12	-1.24	-0.76	<.0001
Hospital Location & Teaching Status					
Rural or Urban Non-teaching	Referent				
Urban Teaching	0.76	0.08	0.59	0.92	<.0001

### Complications                                                                          

**Figure 1:**
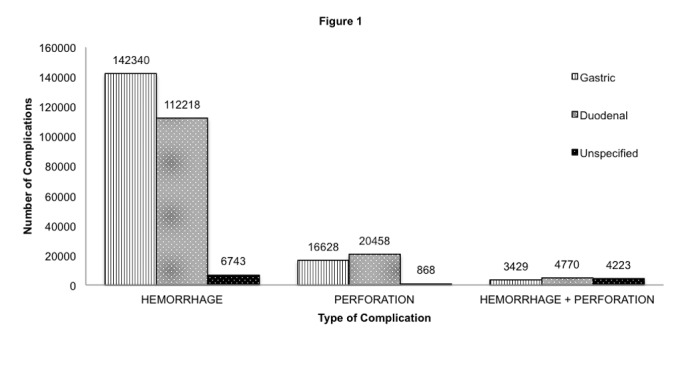
Incidence of peptic ulcer disease complications according to the location of occurrence

Overall total of 311,677 (89%) complications were reported, of which 261,301 (84%) (p <0.0001) were hemorrhages, 37,954 (12%) (p <0.0001) were perforation and 12,422 (four percent) (p<0.0001) included hemorrhage with perforation. As shown in Figure 1, hemorrhage mostly occurred with gastric ulcers (54%), whereas perforations were mostly seen with duodenal ulcers (54%). The likelihood of hemorrhage along with perforation was also higher in duodenal ulcers (38%) when compared to gastric ulcers (28%), and the remaining 34% did not have a specified ulcer location. The rate of major complications like hemorrhage decreased by 66%, perforation by 53.4% and hemorrhage with perforation by 73.7% from 2000 to 2011 (Table 4).

**Table 4:** Trend of PUD complications

	Hemorrhage according to type of ulcer												
	2000	2001	2002	2003	2004	2005	2006	2007	2008	2009	2010	2011	Total
Gastric	19842	18790	14892	14728	13934	12212	10841	8839	8023	7216	6340	6684	142340
Duodenal	16780	14843	11337	11317	10388	8659	8050	7033	6498	6140	5388	5787	112218
Unspecified	1009	894	758	739	660	492	403	429	368	367	305	319	6743
Total	37630	34526	26987	26783	24981	21364	19294	16301	14889	13723	12033	12791	261301
	Perforation according to type of ulcer												
	2000	2001	2002	2003	2004	2005	2006	2007	2008	2009	2010	2011	Total
Gastric	1889	1933	1607	1594	1550	1528	1168	1132	1071	1020	1162	974	16628
Duodenal	2767	2508	2070	1873	1728	1644	1531	1530	1258	1162	1218	1169	20458
Unspecified	104	118	87	62	102	63	61	41	35	60	62	74	868
Total	4760	4559	3764	3529	3380	3235	2760	2703	2364	2243	2442	2217	37954
	Hemorrhage with perforation according to type of ulcer												
	2000	2001	2002	2003	2004	2005	2006	2007	2008	2009	2010	2011	Total
Gastric	431	485	351	383	282	341	337	187	122	223	180	106	3429
Duodenal	641	686	529	538	393	384	275	274	284	298	266	203	4770
Unspecified	749	565	452	422	334	246	430	261	256	113	225	169	4223
Total	1821	1735	1333	1343	1009	971	1042	723	663	634	671	478	12422

 

### Mortality                                                                                   

The overall in-hospital mortality was 4.0% (p < 0.0001). This rate significantly decreased from 4.3% in 2000 to 3.1% by 2011.

### Seasonality of PUD hospitalization in the US and its regions

**Figure 2:**
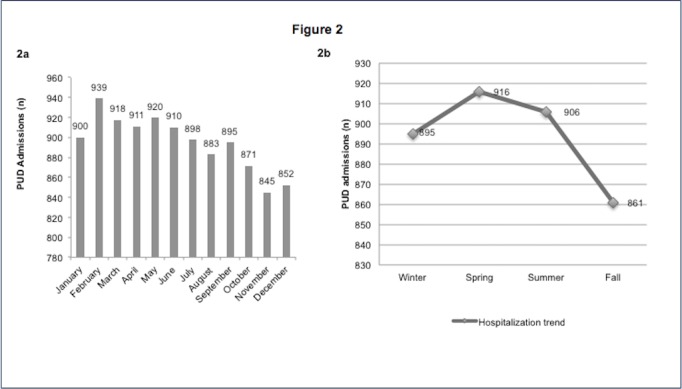
Monthly/seasonal variation of PUD in the United States from 2000 to 2011 2a: Monthly variation of PUD in the United States from 2000 to 2011 2b: Seasonal trends of PUD in the United States from 2000 to 2011

Figure 2a depicts the monthly variation of PUD admissions in the US in the period 2000–2011. We observed two peaks; one in the month of February with 939 per day and the other in May with 920 per day. The month of November had the lowest number of admissions at 845 per day. The seasonal hospitalization trends as shown in Figure 2b translates into a rise in the PUD admissions from winter (895/day), reaching a peak in spring (916/day), and then a gradual decline reaching a nadir in the fall (861/day).

**Figure 3:**
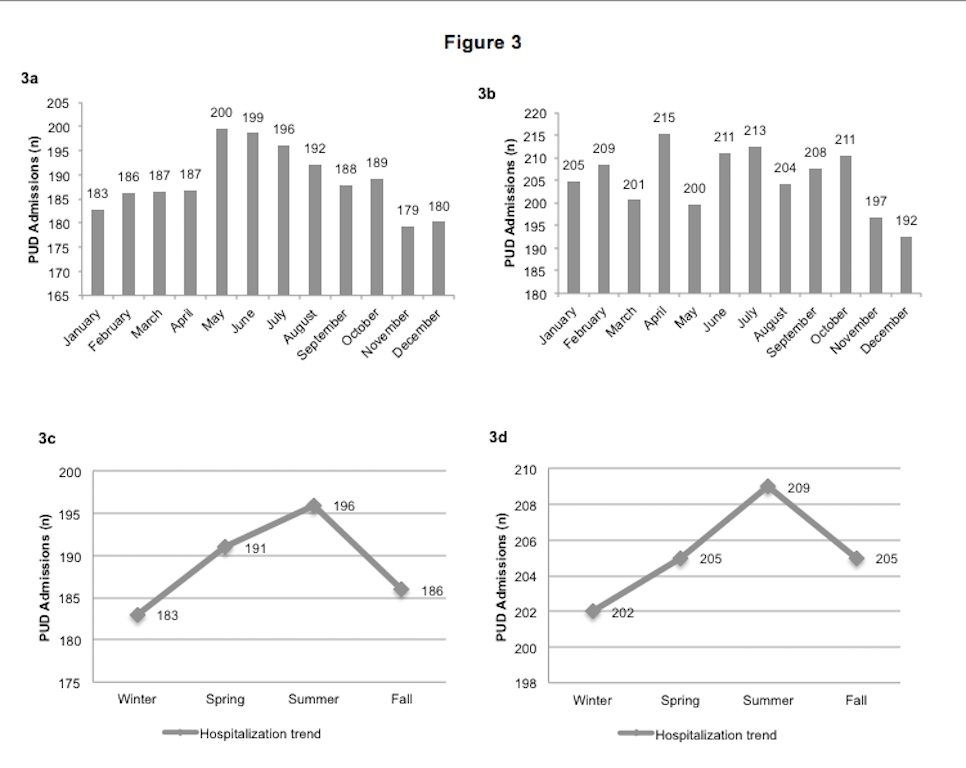
Monthly/ seasonal variation of PUD admissions for Northeast and Midwest Regions from 2000 to 2011 3a: Monthly variation of PUD in the Northeast from 2000 to 2011 3b: Monthly variation of PUD in the Midwest from 2000 to 2011 3c: Seasonal trends of PUD in the Northeast from 2000 to 2011 3d: Seasonal trends of PUD in the Midwest from 2000 to 2011

Figure 3 shows the monthly/seasonal variation of PUD admissions in the Northeast and the Midwest regions. The highest number of admissions for PUD in the Northeast region (Figure 3a) occurred in the month of May (200 admissions/day), followed closely by June (199/day) and lowest in the month of November (179/day). The Midwest region (Figure 3b) had the highest number of admissions for PUD occurring in the months of April (215/day), June (211/day), July (213/day) and October (211/day). In contrast, the fewest number of admissions occurred in the month of December (192/day). The seasonal trend of PUD in the Northeast (Figure 3c) and the Midwest (Figure 3d) regions translated into a gradual rise in admissions from winter (NE (183/day); MW (202/day)) through spring (NE (191/day); MW (205/day)), reaching a peak in summer (NE (196/day); MW (209/day)) and then falling to a low in the fall (NE (186/day); MW (205/day)).

**Figure 4:**
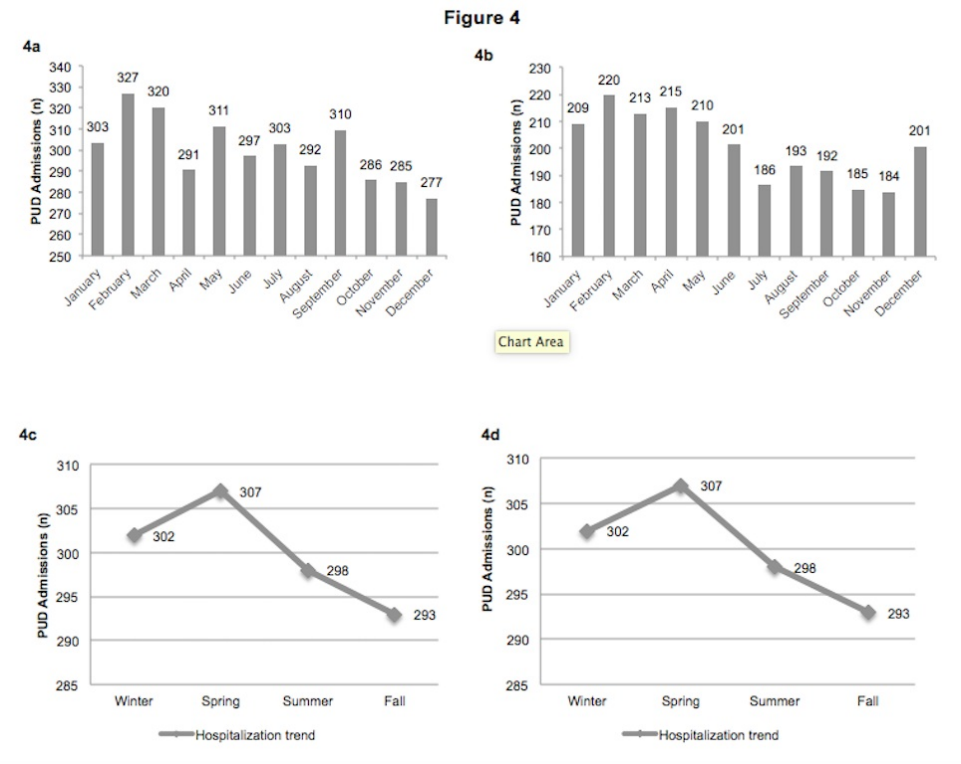
Monthly/ seasonal variation of PUD admissions for South and West Regions from 2000 to 2011 4a: Monthly variation of PUD in the South from 2000 to 2011 4b: Monthly variation of PUD in the West from 2000 to 2011 4c: Seasonal trends of PUD in the South from 2000 to 2011 4d: Seasonal trends of PUD in the West from 2000 to 2011

Figure 4 shows the monthly/seasonal variation of PUD admissions in the Southern and the Western regions. As shown in Figure 4a, the highest number of admissions for PUD in the South occurred in the months of February (327/day), March (320/day), May (311/day) and September (310/day), while the lowest number of admissions occurred in the month of December (277/day). Figure 4b shows the highest number of admissions for PUD in West occurred in the months of February (220/day), March (213/day), April (215/day) and May (210/day) whereas the lowest number of admissions occurred in the month of November (184/day). The seasonal trend of PUD in the Southern region (Figure 4c) and the Western region (Figure 4d) translated into a gradual increase in the number of PUD admissions from winter (S (302/day); W (209/day)), reaching a peak in spring (S (307/day); W (213/day)), falling through summer (S (298/day); W (194/day)) and then reaching a nadir in the fall (S (293/day); W (187/day)).

## Discussion

### Demographics

When assessed, NIS hospitalization trends for PUD in the US over the 12-year period observed an overall decrease in the rate of hospitalizations. This is consistent with the trends reported in prior studies [1, 14]. These findings are possibly due to the increased awareness and treatment of Helicobacter pylori and the usage of proton pump inhibitors (PPIs). The number of PUD admissions was more in males (52.4%) than females (47.6%), which was consistent with other studies that observed PUD to have male sex predominance [4]. This could be due to frequent usage of antimicrobials to treat other illnesses in women [15] and also due to differences in the lifestyle and cigarette smoking pattern between the two sexes [4].

Increased rate of hospitalization for PUD was seen in patients aged 50–64 years. Old age has been associated with a higher risk of PUD due to reduced levels of prostaglandins in the antrum, post-bulbar duodenum and the fundus; thus, exposing the gastric mucosa to a greater risk of ulcerogenic damage [16]. Another possible contributing factor is the increased use of non-steroidal anti-inflammatory drugs (NSAIDs) in the elderly and other concomitant diseases [16].

In our study, PUD admissions were predominantly seen in Whites, which is consistent with the study by Chung, et al. [16] which stated that the ethnic differences may be due to the genetic changes in the gastric mucin and there could be a role of the HLA-DQA1*0102 allele in regards to susceptibility to H. pylori infection. Also, enzymes responsible for slow acetylation, reduced metabolism of debrisoquine and phenytoin play a role in increased PUD incidence and have been found to be about 50% polymorphically distributed in the US population [4].

Variation in lifestyle, socioeconomic factors such as birth outside United States, less education, lower income, household crowding and unknown genetic factors may contribute to the ethnic differences observed in the incidence of PUD [15-16].

### Complications and mortality

Uncomplicated PUD has a lower incidence because of the effective eradication regimens for H. pylori; however, an increasing incidence of the complications related to PUD has been observed [17]. Most of these complications have been attributed to the frequent use of NSAIDs in the US [18]. The most common complication encountered in our study was hemorrhage (84%) mostly in the gastric region (54%). In the United States, gastrointestinal bleeding, resulting from PUD is a major cause of hospitalizations [19]. However, our study shows that there has been a considerable decrease in the number of hospitalizations resulting from hemorrhage from 37,630 in the year 2000 to 12,791 in 2011 (p<0.0001). Similar trends were seen in the of hospitalizations due to perforation and hemorrhage with perforation in both gastric and duodenal sites during the 12-year study period, suggesting better clinical management with revolutionary endoscopic techniques as well as medical therapies like proton pump inhibitors and H-2 receptor antagonists.

The PUD related in-hospital mortality in our study, decreased significantly over the years.

A study by Lewis, et al. [20] suggested that the decreasing mortality and morbidity rates may be due to the increased use of anti-ulcerants rather than decreased NSAID utilization. Expanded indications for diagnostic and screening endoscopy could be another reason for the reduction in the mortality of PUD.

### Cost associated with PUD

Per-capita public health spending (inflation-adjusted) rose from $39 in 1960 to $281 in 2008 and has fallen by 9.3% since then. Public health's share of total health expenditures rose from 1.36% in 1960 to 3.18% in 2002, then fell to 2.65% in 2014; it is projected to fall to 2.40% in 2023 [21]. One of the studies which looked into the direct costs of digestive diseases in the US including inpatient and outpatient services showed PUD to be the fourth most expensive GI disorder [22], which is also reflected by the growing inhospital cost as seen in our study. PUD is the most expensive acid-related disorder due to increased hospitalization especially in the first six months of diagnosis [23].

### Seasonal variation of PUD in the US

Fares, et al. [24] in their study suggest that winter months had a higher incidence of PUD when observed globally. Similar inferences were drawn in other studies [9, 25-26] wherein, the highest number of gastric and duodenal ulcers and related complications were diagnosed between October to March, i.e end of fall and early spring.

Contrary to the strong theory of winter being the peak seasons for peptic ulcers, our study showed that the incidence of PUD admissions in the US started to rise in the winter but peaked in the spring and reached a nadir in the fall. The seasonal variation of PUD admissions in the four regions of the US differed to some extent with the Northeast and Midwest regions having peaks in spring and summer months with a subsequent decline seen in the fall; while the West and South regions had their peaks in spring with a subsequent decline over the summer and reached a nadir in the fall. This may be due to the unique seasonal pattern in each of these regions as the Northeast and the Midwest regions of US experience longer and more severe winters and shorter summers when compared to the South and the West US regions where the summers last longer and the winters are less severe.

The trend of PUD admissions for all four regions started to rise in winter. Xirasagar, et al. [27] have discussed the complex relation of temperature and humidity with the occurrence of duodenal ulcers. They also stated that there is an adverse independent relationship of humidity with older patients and that the seasonality of duodenal ulcers could be generalized in older age groups. Liu, et al. [28], in their study in Nanning, China concluded that there existed a relationship between the meteorological factors and the onset of peptic ulcers such that the winter and the spring season were predominant over summer and fall, supporting our study. Another study concluded that hemorrhages due to gastric ulcers were largely seen in winter months, when the mean temperature and mean vapor pressure were low, while the mean atmospheric pressure was high, contrary to summer when there was a high mean temperature and mean vapor pressure, while the mean atmospheric pressure was low, thus lower incidence [29].

Environmental stress is also a risk factor to PUD. During the colder months, as a result of harsh cold conditions and constantly fluctuating temperatures, the human body experiences considerable acute stress actions triggered by sympathetic nerve excitation and rapid secretion of noradrenaline and adrenaline resulting in the contraction of blood vessels and the duodenal mucosa ultimately leading to mucosal damage due to insufficient oxygen [24]. Thus, the gastric secretions, with high amounts hydrochloric acid, further increase the susceptibility of the damaged mucosa to PUD [24]. The seasonal photoperiodicity invokes the circannual variations of melatonin, which may play a major role in causing duodenal ulcers in addition to the stress caused by harsh winter conditions [27].

In addition to meteorological factors, the seasonal variation observed in our study could also be due to the fact that most adults are prone to worsening of existing conditions like osteoarthritis and rheumatoid arthritis during winter months, which results in the increased NSAID usage [24]. A study suggested that smoking was more common in winters [27]. Other less obvious contributing factors may be due to the presence of concomitant diseases such as Crohn’s disease, Zollinger–Ellison syndrome [18]. Alcohol and caffeine consumption may also contribute, but their association with the seasons is weakly linked and requires further studies.

Our study had certain limitations. The NIS is a retrospective database using administrative ICD 9 codes, thus questioning the accuracy of coding procedures. NIS does not capture readmission rates; hence, the mortality rate could be underestimated. We were unable to directly evaluate the correlation of H. pylori, NSAID exposure, smoking, alcohol intake, caffeine or any other medication use with hospitalization. We could not study independent meteorological factors for the seasonality of PUD. The cost of the disease may be underestimated due to the limitation of measurement of indirect cost such as loss from workdays while consuming health care and the inability to account for long-term care cost like nursing home care. Our data does not include the outpatient encounters and the cost involved in outpatient medications. However, these limitations are counterbalanced by the large sample size and absence of reporting bias as in some publications from specialized centers.

## Conclusions

We noted a decreasing incidence of PUD as well as its complications over a 12-year period. Nevertheless, the rising cost is still a concern that needs to be addressed by developing effective and economical diagnostic or treatment strategies and increasing awareness of the other potential risk factors for PUD. We also noted a seasonal variation of PUD admissions throughout the various regions of the US, with a peak in the spring and nadir in the fall. The variation observed may be due to the different geographical location of the four regions, the effect of meteorological factors on the human body, and the increasing use of NSAIDs in the United States. This variation also has significant economic and clinical implications on our health care system. Being more vigilant and having better availability of hospital resources during this vulnerable period can help to avoid dreaded complications of hemorrhage, perforation, and death. Further large-scale studies focusing on the various risk factors and their association with the seasonal trends of PUD are suggested to better understand the etiology of the disease and thus contributing to its early diagnosis and management.
